# 多发性骨髓瘤患者应用达雷妥尤单抗120 min快速输注方案的安全性和可行性

**DOI:** 10.3760/cma.j.issn.0253-2727.2023.08.017

**Published:** 2023-08

**Authors:** 天航 王, 蕊 郝, 葆楠 胥, 靓 常, 昭宝 刘, 嘉琳 姚, 雯 王, 文君 解, 文强 严, 志坚 肖, 录贵 邱, 刚 安

**Affiliations:** 中国医学科学院血液病医院（中国医学科学院血液学研究所），实验血液学国家重点实验室，国家血液系统疾病临床医学研究中心，细胞生态海河实验室，天津 300020 State Key Laboratory of Experimental Hematology, National Clinical Research Center for Blood Diseases, Haihe Laboratory of Cell Ecosystem, Institute of Hematology & Blood Diseases Hospital, Chinese Academy of Medical Sciences & Peking Union Medical College, Tianjin 300020, China

多发性骨髓瘤（MM）是一种以克隆浆细胞异常增殖为特征的血液系统恶性肿瘤[1]，好发于中老年人，随着我国人口的老龄化，MM发病率逐年上升[2]。达雷妥尤单抗作为第一个被批准用于治疗MM患者的人源化CD38单克隆抗体药物[3]，目前已被国内外多个权威指南推荐用于复发或难治性MM（RRMM）及新诊断MM（NDMM）[4]。达雷妥尤单抗于2015年获得美国食品和药物管理局（FDA）批准，2019年在中国获批上市，随着2021年正式被纳入《国家基本医疗保险、工伤保险和生育保险药品目录（2021年）》，患者的用药需求激增。与其他单克隆抗体类似，输液相关不良反应（IRR）是接受达雷妥尤单抗治疗患者最常见的不良事件（AE），包括上呼吸道症状（咳嗽、咽喉部不适、鼻塞、喘息或呼吸急促）、寒战、皮疹和胃肠道症状等。SIRIUS和GEN501数据的综合分析表明，大多数（95.8％）IRR发生在第1次输液期间，第2次（7％）及以后（7％）IRR发生率较低[5]。目前临床中多按药品说明书的推荐输注速度进行缓慢输注，即首次输注中位时间为7 h，第二次输注中位时间为4.3 h，所有后续输注中位时间为3.5 h[3]。由于当前达雷妥尤单抗输注时间较长且输注频率较高，现有住院资源已不能满足用药需求，亟需验证更短时间输注方案的安全性，探索门诊或日间病房输注的可行性。

基于既往国外关于缩短输液时间的可行性和安全性的报道[6]–[8]，本中心尝试探索达雷妥尤单抗的快速输注方案。由于MM以老年患者居多，且常伴心脑血管基础疾病，考虑到国外90 min快速输注方案较快的输液速度可能造成循环负荷，我们在满足日间病房输液需求的前提下，尝试了一种新的120 min快速输注方案，并进行前瞻性临床研究，评估该快速输注方案的安全性及可行性，现将本临床研究结果报道如下。

## 病例与方法

1. 病例：本研究为单中心前瞻性临床研究。纳入2022年3月14日至2022年7月6日在中国医学科学院血液病医院淋巴瘤诊疗中心就诊并接受过两次及以上达雷妥尤单抗输注的MM患者。纳入标准：①既往接受过≥2次达雷妥尤单抗输注；②既往未发生过3级及以上IRR；③已签署知情同意书。排除标准：①既往达雷妥尤单抗治疗期间发生≥3级IRR；②纽约心功能分级（NYHA）Ⅲ、Ⅳ级患者；③研究者认为不适合入组或因其他原因可能不能完成本试验者。本研究已通过中国医学科学院血液病医院伦理委员会批准（IIT2020023-EC-1），并获得患者的知情同意。

2. 120 min快速输注方案：所有入组患者在前2次达雷妥尤单抗液体输注过程中均按说明书的输注要求进行输注，并可耐受200 ml/h的输液速率。第3次及以后的输注在不改变推荐剂量及液体浓度的前提下采用了120 min快速输注方案，与国外的90 min方案比较，最大输注速率显著降低，与标准输注方案比较，总输注时长有明显优势（表1）。在120 min快速输注方案中仍使用达雷妥尤单抗的推荐剂量16 mg/kg，配制时保持稀释后液体总量为500 ml；初始输注速率为200 ml/h，前30 min输入液体总量的20％（100 ml）；后90 min调整输注速率至270 ml/h，输入剩余80％（400 ml）液体。输注前使用的药物包括地塞米松20 mg、氯苯那敏4～8 mg、异丙嗪25 mg、对乙酰氨基酚0.5～1 g、孟鲁司特10 mg。

3. 不良反应：输注前和输注后30 min测量患者血压，输注中密切观察，记录患者不良反应，1周后对患者进行电话随访，评估有无输注后迟发不良反应。不良反应的分级按照美国国家癌症研究所（NCI）常用不良事件术语标准（CTCAE，版本5.0）进行评估，分为Ⅰ～Ⅳ级。若患者出现3～4级IRR，则立即终止输注，予对症处理，下一次输注改用标准输注方案；若患者未出现3～4级不良输注反应，下一个疗程可继续使用达雷妥尤单抗快速输注方案。

4. 结局指标：研究的主要终点是根据IRR的发生率、类型和严重程度评价达雷妥尤单抗快速输注方案的安全性。次要终点为比较不同年龄和合并症患者IRR的发生情况。

5. 统计学处理：采用SPSS 24.0软件进行统计学分析。采用描述性方法总结人口学特征、临床特征和安全性数据，计数资料采用频数（百分比）描述，计量资料用中位数（范围）或*x±s*描述。采用*t*检验比较快速输注前和输注后30 min的血压，采用Fisher确切概率法比较不同亚组患者不良反应的发生率，*P*<0.05为差异有统计学意义。

**表1:** 达雷妥尤单抗快速输注方案与标准输注方案的比较

方案	总时长	20%剂量（100 ml）			80%剂量（400 ml）	
		输注时长	起始速率（ml/h）		输注时长	最大速率（ml/h）
标准输注	3 h 15 min	1 h	100		2 h 15 min	200
快速输注	120 min	30 min	200		90 min	270
	90 min	30 min	200		60 min	400～450

## 结果

1. 临床特征：共纳入60例MM患者，共于我院日间病房接受达雷妥尤单抗快速输注方案204次，每例患者中位输注3（1～10）次。男43例（71.7％），女17例（28.3％），中位年龄为59（31～77）岁，≥65岁患者20例（33.3％）。5例（8.3％）患者有药物过敏史，2例（3.3％）患者有慢性阻塞性肺疾病（COPD）病史。14例（23.3％）患者有高血压病史，其中4例（28.6％）为高血压3级。9例（15.0％）患者有冠状动脉粥样硬化性心脏病病史，16例（26.7％）患者肾功能不全（内生肌酐清除率<80 ml/min）。28例（46.7％）患者无基础疾病。所有患者均接受以达雷妥尤单抗为基础的联合治疗，其中29例（48.3％）接受达雷妥尤单抗+硼替佐米+来那度胺+地塞米松方案，13例（21.7％）接受达雷妥尤单抗+来那度胺+地塞米松方案，5例（8.3％）接受达雷妥尤单抗+硼替佐米+地塞米松方案，2例（3.3％）接受达雷妥尤单抗+硼替佐米+泊马度胺+地塞米松方案，2例（3.3％）接受达雷妥尤单抗+硼替佐米+环磷酰胺+地塞米松方案，9例（15.0％）接受其他方案。10例（16.7％）患者在第3次输注时开始使用快速输注方案，19例（31.7％）患者在第4～8次输注时开始使用快速输注方案，31例（51.7％）患者在第9次及以后输注时开始使用快速输注方案。13例患者（21.7％）在第1次输注达雷妥尤单抗时发生过IRR，仅1例在随后的快速输注中再次发生IRR。

2. IRR：快速输注组有3例（5％）患者发生了IRR。例1表现为眼睛发麻、脸部瘙痒，为2级IRR，IRR发生时输液速率为200 ml/h。应用甲泼尼龙后可迅速缓解，缓解后按起始速率100 ml/h继续输注剩余达雷妥尤单抗液体，最快速率可达270 ml/h。该患者因自体造血干细胞移植而中断达雷妥尤单抗治疗6个月，此次为移植后第1次输注，患者此前已经按标准输注方案输注过12次，除第1次外均未发生IRR。例2在输注30 min后出现血压降低，未再增加输注速率，以起始速率200 ml/h输注完毕，为1级IRR。例3快速输注期间未发生不良反应，在随访过程中发现回家后出现腹泻症状，对症口服蒙脱石散后缓解。以上患者在后续接受达雷妥尤单抗快速输注方案时均未再次发生IRR。其余患者未发生任何级别的IRR。快速输注前的平均收缩压［（124.23±15.91）mmHg，1 mmHg＝0.133 kPa］和输注后30 min的平均收缩压［（122.65±15.21）mmHg］相比，差异无统计学意义（*P*＝0.074），快速输注前和输注后30 min的平均舒张压差异亦无统计学意义［（88.22±9.34）mmHg对（79.00±9.99）mmHg，*P*＝0.081］。

<65岁组患者40例，2例（5％）发生IRR，≥65岁组患者20例，1例（5％）发生IRR，两组IRR发生率的差异无统计学意义（*P*＝1.000）。既往标准输注时出现IRR的患者13例，1例（7.8％）在快速输注时发生IRR，既往标准输注时未出现IRR的患者47例，2例（4.3％）在快速输注时发生IRR。有基础疾病组（COPD、高血压、冠状动脉粥样硬化性心脏病、肾功能不全）患者32例，1例（3.1％）发生IRR，无基础疾病组患者28例，2例（7.1％）发生IRR，两组患者IRR发生率的差异无统计学意义（*P*＝0.601）。

## 讨论

MM是血液系统第二大恶性肿瘤，多种新药如蛋白酶体抑制剂、免疫调节剂等的应用可有效改善患者预后，但MM目前仍无法治愈。达雷妥尤单抗作为首个批准用于治疗MM患者的CD38单克隆抗体，已成为免疫治疗时代下MM治疗的重要基石，多项临床试验证实单药或联合应用达雷妥尤单抗治疗MM患者可取得极佳的疗效。但考虑到当前达雷妥尤单抗标准输注时间较长且用药频率较高，达雷妥尤单抗快速输注可实现医疗资源的最大利用，促进我国MM患者治疗模式转变。

2018年国外提出达雷妥尤单抗90 min快速输注方案[6]，其输液速度高达400～450 ml/h，按照输液器滴系数20滴/ml进行计算，为134～150滴/min，远高于我国护理学教材中要求的标准输液速度[9]。同时，MM好发于老年人，且许多MM患者合并肾功能不全、心功能不全，标准输液速度应当适当减慢。基于此，我们探索了一种120 min快速输注方案，最快速率为270 ml/h（90滴/min），既能满足门诊和日间病房对输注时间的需求，也更贴近我国的输液规范。根据既往研究报道的达雷妥尤单抗快速输注的不良反应发生率，如果IRR的发生率低于5.3％（95％*CI* 2.7％～10.2％），则认为该方案是安全的[10]。在本研究应用达雷妥尤单抗快速输注方案的患者中仅3例（5％）发生IRR，且均为1～2级，与国外其他研究的结果一致[6],[11]。3例患者发生的IRR均可控，且未影响达雷妥尤单抗的后续输注，说明该120 min输注方案具有足够的安全性和可行性。

值得注意的是，1例患者因自体造血干细胞移植中断达雷妥尤单抗治疗6个月，再次应用快速输注方案后出现IRR，主要表现为眼睛发麻、脸部瘙痒，未出现红斑、皮疹。相似的情况也发生在Bonello等[10]的研究中，有部分患者中断达雷妥尤单抗治疗2个月，其中22％的患者再次应用快速输注方案后出现IRR。在GRIFFIN和CASSIOPEIA试验中，有3％和11％的患者在自体造血干细胞移植后恢复使用达雷妥尤单抗时出现IRR[12]–[13]。因此，治疗中断后再次恢复治疗可能是发生IRR的主要原因，与输注速度的相关性较小，提示为中断后再次恢复达雷妥尤单抗治疗的患者输注时，应按首次输注要求执行，避免采用快速输注方案，以减少IRR的发生。同时，本研究中13例首次输注达雷妥尤单抗出现IRR的患者中仅1例在快速输注时再次出现IRR，提示无论首次输注是否出现IRR，可耐受200 ml/h输液速度的MM患者均可应用达雷妥尤单抗快速输注方案。

本研究排除了心功能Ⅲ、Ⅳ级的患者，但仍有7例入组患者存在继发性淀粉样变性（累及心脏/肾脏），14例患者合并1～3级高血压，达雷妥尤单抗快速输注前后的血压值差异无统计学意义，16例患者内生肌酐清除率<80 ml/min，所有患者在快速输注后均未出现循环负荷过重表现。研究显示，不同年龄组和有无基础疾病组患者快速输注后IRR发生率的差异均无统计学意义（*P*值均>0.05），以上结果为伴基础疾病的MM患者应用快速输注方案提供了参考。与本研究结果类似，Hamadeh等[14]的回顾性队列研究中也有3例淀粉样变性患者和6例伴有基础疾病（心力衰竭、哮喘和COPD）的患者接受了达雷妥尤单抗快速输注方案，均未出现明显不良反应，提示伴器官功能障碍的MM患者不应作为快速输注方案的排除标准。

为更好地保障患者应用达雷妥尤单抗快速输注方案期间的安全性，我们根据欧洲肿瘤内科学会（ESMO）发布的《系统性抗癌治疗输液反应的管理指南》[15]制定了IRR应急处理流程：①怀疑发生输液反应时，立即停止输注药物，输注生理盐水，保持血管通路。②安慰患者，减轻患者的心理焦虑。③评估患者气道、呼吸和循环系统的状态，必要时予氧气吸入；评估意识水平；测量生命体征；观察患者的皮肤变化，有无荨麻疹、血管神经性水肿和红斑。④根据患者症状，遵医嘱予组胺受体阻滞剂、糖皮质激素及退热剂进行对症治疗，备好抢救仪器。⑤协助患者取合适体位。⑥在症状完全消失后，以50％的速度输注并逐渐增加至患者可耐受剂量。⑦24 h内密切观察患者的生命体征。

本研究为单中心的单臂前瞻性临床研究，未设置对照组，且样本量较小，但作为我国首个前瞻性达雷妥尤单抗快速输注临床研究，为120 min快速输注方案提供了一定的临床证据，具有一定的临床应用价值，值得进一步在多中心开展大样本对照研究，进一步评估120 min快速输注方案的安全性和可行性。

综上所述，达雷妥尤单抗120 min快速输注方案应用于接受两次及以上达雷妥尤单抗输注的MM患者，不良反应发生率低。不同年龄、有无基础疾病对于MM患者应用快速输注方案时的IRR发生率并无显著影响。本研究证实达雷妥尤单抗120 min快速输注方案可满足患者在日间病房或门诊输注的需求，较标准方案提高床/椅使用率，最大限度地合理分配医疗资源。
